# Inhibin A—A Promising Predictive Parameter for Determination of Final Oocyte Maturation in Ovarian Stimulation for IVF/ICSI

**DOI:** 10.3389/fendo.2020.00307

**Published:** 2020-05-15

**Authors:** Barbara Lawrenz, Leyla Depret Bixio, Carol Coughlan, Claus Yding Andersen, Laura Melado, Bhanu Kalra, Gopal Savjani, Human M. Fatemi, Ajay Kumar

**Affiliations:** ^1^IVF Department, IVIRMA Middle-East Fertility Clinic, Abu Dhabi, United Arab Emirates; ^2^Department of Obstetrical, Women's University Hospital Tuebingen, Tübingen, Germany; ^3^Laboratory of Reproductive Biology, The Juliane Marie Centre for Women, Children and Reproduction, Copenhagen University Hospital and Faculty of Health and Medical Sciences, University of Copenhagen, Copenhagen, Denmark; ^4^Ansh Labs, Webster, TX, United States

**Keywords:** Inhibin A, Inhibin B, estradiol, ovarian stimulation, oocyte maturity

## Abstract

The number of mature oocytes is a key factor in the success of Assisted Reproductive Techniques (ART). Exogenous gonadotropins are administered during ovarian stimulation in order to maximize the number of oocytes available for fertilization. During stimulation, monitoring is mandatory to evaluate individual response, to avoid treatment complications and assist in the determination of the optimal day for final oocyte maturation and oocyte retrieval. Routine monitoring during stimulation includes transvaginal ultrasound examinations and measurement of serum estradiol (E2). Due to multifollicular growth of follicles of varying size, serum E2 levels are commonly supraphysiological and often variable, rendering E2-measurement during ovarian stimulation unreliable as a determinant of oocyte maturity. In contrast to serum E2, serum Inhibin A levels increase once a minimum follicle size of 12–15 mm is achieved. Due to this fact, serum Inhibin A levels could present in combination with ultrasound monitoring a more reliable parameter to determine the optimal follicle size for final oocyte maturation, as only follicles with a size of 12 mm and beyond will contribute to the serum Inhibin A level. This prospective observational, cross-sectional study demonstrates, that on the day of final oocyte maturation serum Inhibin A is strongly correlated to the number of follicles ≥15 mm (0.72) and to the number of retrieved and mature oocytes (ρ 0.82/0.77, respectively), whereas serum E2 is moderately correlated to the parameters mentioned above (ρ 0.64/0.69/0.69, respectively). With an area under the curve (AUC) of 0.91 for Inhibin A, compared to an AUC of 0.84 for E2, Inhibin A can be regarded as a better predictor for the optimal timing of trigger medication with a threshold number of ≥10 mature oocytes. It can be concluded from this data that serum Inhibin A in combination with transvaginal ultrasound monitoring may be a more powerful tool in the decision making process on trigger timing as compared to E2.

## Introduction

The number of retrieved oocytes is critical to the success of IVF treatment. As a result, the aim of ovarian stimulation for IVF is to maximize the number of oocytes available for fertilization (1, 2). In order to achieve this goal, ovarian stimulation prior to IVF requires the administration of exogenous gonadotropins to support multi-follicular growth until the day of final oocyte maturation (3). Currently, ultrasonographic determination of the antral follicle count (AFC) and/or the measurement of Anti-Müllerian-Hormone (AMH) prior to stimulation start assist in the determination of the optimal gonadotropin dose to prescribe, the identification of patients at risk of developing ovarian hyperstimulation syndrome (OHSS) (4) or a low/no response during stimulation (5). Despite the assessment of these parameters as an indicator for the expected treatment response prior to cycle initiation, close monitoring of individual response to ovarian stimulation is mandatory to avoid treatment complications, facilitate individualization of treatment and assist in the determination of the optimal day for final oocyte maturation and oocyte retrieval.

Routine monitoring of ovarian stimulation for IVF/ICSI includes transvaginal ultrasound examinations (TVUS) and measurement of serum estradiol (E2) (6). With TVUS, ovarian response to gonadotropin administration is monitored by recording the size and number of developing antral follicles and serum E2 levels that reflect the collective hormonal capacity of the follicles.

In a natural cycle, aromatase activity begins to increase on cycle day 5–8 in follicles larger than 8 mm (7, 8). Upon selection, the dominant follicle in a natural cycle produces more E2 than the other follicles and the E2 level increases with the increasing size of the dominant follicle.

Whereas, in a natural cycle the serum E2 level can give an indication of follicle size and the maturation process of the oocyte, serum E2 levels in ovarian stimulation cycles are supraphysiological and often variable due to growth of multiple follicles of varying size. Therefore, E2-measurement is not reliable in ovarian stimulation cycles as the sole determinant when choosing the optimal time for administration of trigger medication. In contrast to E2, Inhibin A levels increase from a follicle size of 12–15 mm and beyond (9–11). As only follicles from these sizes onwards will contribute to the serum Inhibin A levels, the combination of serum Inhibin A measurement and TVUS could present a more reliable parameter for determining the optimal timing for administration of final oocyte medication, as compared to serum E2 plus TVUS.

Previous publications have described Inhibin A levels during stimulation (11) and correlated these levels with ART outcome (12). To date the potential of serum Inhibin A as a decision-making tool in determining the optimal timing of final oocyte maturation has never been studied.

Therefore, the aim of this prospective observational, cross-sectional study was to evaluate the role of serum Inhibin A as predictor of the number of retrieved and mature oocytes as compared to serial measurements of E2 in a routine setting of a private IVF center and to define a cut-off level of serum Inhibin A above which retrieval of ≥10 mature oocytes is likely. This study will determine if serum Inhibin A could potentially serve as decision making tool meriting inclusion of Inhibin A into routine cycle monitoring.

## Materials and Methods

This observational prospective, cross-sectional study was performed in IVIRMA Fertility Clinic, Abu Dhabi, UAE, between September 2018 and January 2019. All patients, independent of the AFC as a quantitative marker of the ovarian reserve, undergoing ovarian stimulation for IVF/ICSI due to primary or secondary infertility during this time period in a GnRH-antagonist-protocol and who consented to take part in this study, were included. Only one stimulation cycle was included from each patient.

On day 2 or 3 of the period of the planned treatment cycle, prior to initiation of stimulation, a vaginal ultrasound was performed to determine the AFC. All follicles with a diameter between 2 and 10 mm in each ovary were recorded and the numbers added to determine the total AFC-count (13). In keeping with routine clinical practice patients were monitored during ovarian stimulation for IVF/ICSI treatment with serial transvaginal ultrasound examinations. Transvaginal scans were performed using a Voluson 6 (GE Healthcare, Milwaukee, WI, USA) ultrasound machine, equipped with a 7–10 MHz two-dimensional transvaginal probe. The patients were asked to empty their bladders and were placed in the lithotomy position.

During ovarian stimulation, patients were seen two or three times for ultrasound monitoring of follicular response and serum hormonal measurement in accordance with ovarian response.

Follicle size and number was determined during the course of stimulation and on the day of final oocyte maturation by vaginal ultrasound, as previously described. Follicle diameter was determined by measuring two orthogonal diameters and the mean value was recorded as follicle size.

Blood samples for this study were taken in addition to routine hormonal measurements (FSH, LH, E2 and progesterone), used for stimulation monitoring, on cycle day 2 or 3 prior to initiation of stimulation and on the day of final oocyte maturation. The blood was centrifuged for 10 min at 2,688 g (relative centrifugal force) per minute and the supernatant was retrieved and frozen at −21°C. For the measurement of serum levels of E2, Inhibin A and Inhibin B, the samples were thawed by keeping them for maximum 90 min at room temperature (~20–24°C) and analyzed the same day with the same batch of reagents.

The following demographic data per patient were recorded: age, Body Mass Index (BMI), number of infertility years and number of previous stimulations. On the day of final oocyte maturation, the total number of follicles, the number of follicles <15 and ≥15 mm and the cycle day were registered. Moreover, the number of stimulation days, the total gonadotropin dose required and the number of retrieved and mature oocytes were recorded.

### Ovarian Stimulation Protocols

Ovarian stimulations were performed in Gonadotropin-Releasing-Hormone (GnRH)-antagonist-protocols, using recFSH (recombinant Follicle-stimulating-hormone) or human-Menopausal-Gonadotropin (HMG) as stimulation medication. Stimulation medication dosage was individualized prior to stimulation start in accordance with the quantitative parameters, reflecting ovarian reserve (14). During ovarian stimulation, medication dose was adjusted in line with ovarian response as determined by ultrasound scan findings and measured serum levels of E2 and progesterone (P4). In order to avoid P4 elevation during the late follicular phase a reduction in medication dose may have been warranted as determined by serial monitoring of progesterone levels (15). Final oocyte maturation was achieved by administration of either 10.000 IU of hCG, 0.3 mg of GnRH agonist (Triptorelin) or dual trigger (hCG and GnRH-analog), as soon as ≥3 follicles ≥17 mm were present, depending on the ovarian response and clinician discretion. In low responders with <3 growing follicles, medication for final oocyte maturation was given when at least 1 follicle of ≥17 mm was present. Oocyte retrieval was carried out 36 h after administration of the trigger.

Ultrasound findings and serum E2 levels formed the basis on which decisions were taken regarding cycle monitoring and trigger timing. The blood samples, obtained for study-purposes, were later analyzed for serum levels of Inhibin A, B and again for E2 in order to avoid bias through batch-to-batch inconsistencies for the herein presented analysis.

### Hormonal Analysis

#### Inhibin A Analysis

Commercially available hypersensitive and specific immunoassays from Ansh Lab, Texas, USA were used to detect levels of Inhibin-A (AL-123). All samples were run neat in Inhibin A ELISA. Samples reading higher than the highest Calibrator in the assay were diluted 1:10 in calibrator A/sample diluent of the kit and re-assayed. The coefficient of variation for the Inhibin A assay over 10 assay runs for two kit controls at 105 and 348 pg/mL and two serum controls at 47 and 135 pg/mL was 4.7, 3.4, 4.6, and 3.9%, respectively. The calibrators in the Inhibin A ELISA are traceable to WHO reference preparation (WHO 91/624). The traceability factor is reported as slope of observed WHO preparation w.r.t. known concentration when analyzed in the ELISA. Inhibin A = 1.68 (WHO 91/624). The assay is specific to Inhibin A and does not crossreact with closely related analytes such as Inhibin B, Activin A, Activin B, Activin AB, FST-315, and FSTL3 when spiked at 50 ng/mL in analyte free matrix.

The Inhibin A in serum is stable up to 3 freeze thaw cycles. The assay is designed to measure mature Inhibin A and does not detect Activin A and Inhibin alpha fragments.

#### Estradiol Analysis

Estradiol measurements were performed using commercially available kits from DRG International (EIA-2693, DRG ELISA). All samples were run neat. Samples reading higher than that Calibrator F in the assay were diluted 1:10 in the calibrator A/sample diluent of the kit and re-assayed. The coefficient of variation for the E2 assay over 10 assay runs for two spiked E2 controls at 111 and 423 pg/mL and one serum control at 796 pg/mL was 9.8, 3.9, and 6.3, respectively.

#### Inhibin B Analysis

Commercially available hypersensitive and specific Inhibin B immunoassay (AL-107) from Ansh Lab, Texas, USA were used to detect Inhibin-B levels in serum. All samples were run neat in inhibin B ELISA. Samples reading higher than the highest Calibrator in the assays were diluted 1:10 in calibrator A/sample diluent of the kit and re-assayed. The coefficient of variation for the inhibin B assay over 12 assay runs for two kit controls at 126 and 345 pg/mL and two serum controls at 87 and 218 pg/mL was 2.0, 2.3, 3.8, and 4.0%, respectively. The calibrators in the Inhibin B ELISA are traceable to WHO reference preparation. Inhibin B = 0.4 (WHO 96/784, the WHO preparation is a mixture of Inhibin A, Inhibin B, and Inhibin alpha). The assay is specific to Inhibin B and does not cross-react with closely related analytes such as Inhibin A, Activin A, Activin B, Activin AB, AMH, FST-315, and FSTL3 when spiked at 50 ng/mL in analyte free matrix.

The Inhibin B in serum is stable up to 3 freeze thaw cycles. The assay is designed to measure mature Inhibin B and does not detect Activin B and Inhibin alpha fragments.

### Statistical Analysis

Continous data are presented as mean ± SD, 95%CI, minimum and maximum values when appropriate. The assumption of normality was checked using a proc univariate. Pearson's Fisher Z-Transformation test (ρ) was used to test the strength of the correlation between Inhibin A, Inhibin B, E2 serum levels, and different variables at given cycle times. One way ANOVA was used to analyze mean changes on serum levels of Inhibin A and B, E2, and number of follicles, number of retrieved and mature ocytes as category variables. Logistic regression was used to find the Area Under the Curve (AUC) to determine the capacity of Inhibin A and E2 serum levels to predict mature oocytes. ROC curve analysis was performed, using SAS studio™ software, with Inhibin A and E2 as the classifier to predict oocyte maturity. AUC and 95% confidence intervals around the AUC were computed.

To find the optimal treshold for serum Inhibin A level to predict mature oocytes, the Youden index was calculated (Youden index = sensitivity + specificity −1). For this analysis a cut-off of ≥10 mature oocytes was applied. All analyses were performed using SAS studio (Copyright © 2018 SAS Institute Inc., Cary, NC, USA.). For interpretation of the results, a *p* < 0.05 was considered to be statistically significant.

Interpretation of Pearson coefficient was done according to Schober et al. (16):

0.00–0.10 = Negligible correlation
0.10–0.39 = Weak correlation
0.40–0.69 = Moderate correlation
0.70–0.89 = Strong correlation
0.90–1.00 = Very strong correlation

For interpretation of the results, a *p* < 0.05 was considered to be statistically significant.

Interpretation of ROC curve was done according to Li and He (17):

0.90–1 = Excellent
0.80–0.90 = Good
0.70–0.80 = Fair
0.60–0.70 = Poor
0.50–0.60 = Fail.

#### Ethical Approval and Trial Registration Number

This study was approved by the ethics committee of the IVIRMA Abu Dhabi Fertility Clinic, Abu Dhabi, UAE (approval number: REFA019) and was registered with clinicaltrials.gov. under the number NCT03607409.

## Results

Results of blood samples and parameters from the ovarian stimulation treatment for IVF/ICSI were available from a total of 145 patients at the start of ovarian stimulation (cycle day 2/3) and from 136 patients on the day of final oocyte maturation. From nine patients there were no recorded follicle measurements and no blood test results available on the day of final oocyte maturation due to cycle cancellation for various reasons (no/low response, patient's decision to cancel treatment due to personal reasons).

The mean age (±SD) of patients evaluated was 35.4 ± 6.5 years and mean BMI was 28.2 ± 4.8 kg/m^2^ with a history of 3.9 ± 3.0 years of infertility and a mean number of 3.1 ± 4.2 previous stimulations. Patients' characteristics, AFC, the serum levels of Inhibin A, E2, and Inhibin B on cycle day 2 or 3, are summarized in Table 1.

**Table 1:** Patients' characteristics and results of AFC, serum levels of Inhibin A, E2, and Inhibin B on cycle day 2 or 3.

**Parameter**	**Number of samples**	**Mean**	***SD***	**95%CI mean**		**Min**	**Max**
				**Lower bound**	**Upper bound**		
Infertility years	145	3.9	3.0	3.4	4.4	0	16
Previous stimulations	145	3.1	4.2	2.5	3.8	0	26
Age (years)	145	35.4	6.5	34.2	36.3	21	48
BMI (kg/m^2^)	145	28.2	4.8	27.4	28.9	17.5	41.7
AFC (n)	145	12.2	7.3	11.0	13.4	0	30
Inhibin A (pg/ml)	145	7.7	3.9	7.0	8.3	4.9	24.7
E2 (pg/ml)	145	63.2	28.4	58.5	67.8	17.2	219.6
Inhibin B (pg/ml)	145	89.2	65.6	78.5	100.0	1.60	503.0

*BMI, body mass index; E2, estradiol; SD, standard deviation*.

A correlation test was applied in order to obtain the probability values (*p*-value) and the Pearson's coefficient (ρ). For the probability values, a statistically highly significant correlation was found between AFC and Inhibin B (*p* < 0.0001) and statistically no significant correlation between AFC and Inhibin A/E2 (*p*-value 0.16/0.41, respectively). The Pearson's coefficient (ρ) was moderate between AFC and Inhibin B (ρ 0.40; CI95% [0.26; 0.53]), weak/negligible between AFC and Inhibin A/E2 (ρ 0.12; CI95% [-0.04; 0.27]/−0.07; CI95% [−0.22; 0.09]), respectively.

On the day of final oocyte maturation, blood samples for Inhibin A, E2, and Inhibin B measurement were available from 136 patients. The results of hormonal measurements as well of the stimulation parameters and outcomes are listed in Table 2.

**Table 2:** Patients' characteristics and results of AFC, Inhibin A, estradiol, and Inhibin B on the day of final oocyte maturation.

**Parameter**	**Number of samples**	**Mean**	***SD***	**95%CI mean**		**Min**	**Max**
				**Lower bound**	**Upper bound**		
Infertility years (*n*)	136	3.89	3.02	3.38	4.40	0	16
Previous stimulations (*n*)	136	3.21	4.25	2.49	3.93	0	26
Age (years)	136	35.2	6.5	34.0	36.3	21	48
BMI (kg/m^2^)	136	27.9	4.8	27.1	28.8	17.5	41.6
AFC (*n*)	136	12.3	7.4	11.0	13.5	0	30
Inhibin A (pg/ml)	136	827.7	632.9	720.4	935.1	10.6	3859.5
E2 (pg/ml)	136	2105.3	1588.8	1835.9	2374.7	82.9	9005.1
Inhibin B (pg/ml)	136	1706.6	1997.5	137.9	2045.4	17.6	13972.1
Total number of follicles	136	14.24	7.68	12.9	15.5	1	32
Number of follicles <15 mm	136	7.1	5.1	6.3	8.0	0	24
Number of follicles ≥15 mm	136	7.0	5.2	6.1	7.9	1	25
Cycle day of trigger	136	12.1	1.7	11.8	12.4	8	16
Stimulation days (*n*)	136	10.1	1.6	9.8	10.4	6	14
P4 trigger day (ng/ml)	136	0.6	0.4	0.5	0.7	0.05	2.2
Total dosage gonadotropins (IU)	136	3363.7	1218.0	3157.2	3570.3	1125.0	6150.0
Number of retrieved oocytes	136	12.0	9.0	10.5	13.5	0	40
Number of mature oocytes	136	9.1	7.1	7.9	10.3	0	32

*SD, standard deviation; CI, confidence interval; BMI, body mass index; AFC, antral follicle count; E2, estradiol; P4, progesterone*.

All ultrasonographic stimulation parameters (total number of follicles, number of follicles <15 mm, number of follicles ≥15 mm) showed a statistically highly significant correlation with hormonal serum levels of Inhibin A, E2, and Inhibin B (*p* < 0.0001). A strong correlation was found with Pearson's coefficient between the total number of follicles and serum levels of Inhibin A, E2, and Inhibin B (0.78; CI95% [0.70; 0.84]/0.71; CI95% [0.63; 0.79]/0.73; CI95% [0.64; 0.80], respectively) and between the number of follicles ≥15 mm and Inhibin A (0.72; CI95% [0.62; 0.79]). Table 3 lists the correlations.

**Table 3:** Correlations on the day of final oocyte maturation between total numbers of follicles/numbers of follicles <15 mm/numbers of follicles ≥15 mm and Inhibin A, estradiol, and Inhibin B.

**Follicle number on the day of final oocyte maturation**	**Inhibin A**	**E2**	**Inhibin B**
Total number of follicles Pearson coefficient	*p* < 0.0001 0.78 CI95% [0.70; 0.84]	*p* < 0.0001 0.71 CI95% [0.63; 0.79]	*p* < 0.0001 0.73 CI95% [0.64; 0.80]
Number of follicles <15 mm Pearson coefficient	*p* < 0.0001 0.43186 CI95% [0.28; 0.55]	*p* < 0.0001 0.40936 CI95% [0.25; 0.54]	*p* < 0.0001 0.52855 CI95% [0.39; 0.63]
Number of follicles ≥15 mm Pearson coefficient	*p* < 0.0001 0.72 CI95% [0.62; 0.79]	*p* < 0.0001 0.64 CI95% [0.53; 0.73]	*p* < 0.0001 0.55138 CI95% [0.42; 0.65]

*E2, estradiol; CI95%, confidence interval*.

**Figure 1:**
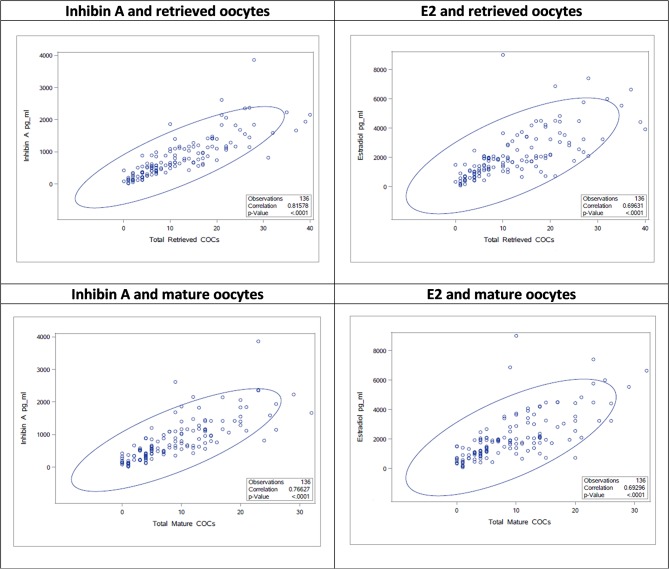
ROC curve analysis of Inhibin A and E2 according to the number of ≥10 mature oocytes.

The stimulation parameters on the day of final oocyte maturation (total gonadotropin dosage, number of retrieved and mature oocytes) were statistically highly significant (*p* < 0.0001) correlated with the results of serum levels of Inhibin A, E2, and Inhibin B. A strong correlation was found between Inhibin A and number of retrieved oocytes and mature oocytes (ρ 0.82; CI95% [0.75; 0.86]/ρ 0.77; CI95% [0.69; 0.83]), E2 showed a moderate correlation (ρ 0.69; CI95% [0.60; 0.77]/ρ 0.69; CI95% [0.59; 0.77]), and Inhibin B a strong/moderate correlation (ρ 0.71; CI95% [0.62; 0.78]/ρ 0.65; CI95% [0.54; 0.74]), respectively. The correlations of the stimulation parameters with Inhibin A, estradiol and Inhibin B are summarized in Table 4 and Figure 1 depicts the correlations between serum Inhibin A and E2 levels and the number of retrieved and mature oocytes.

**Table 4:** Correlations on the day of final oocyte maturation between the stimulation parameters and Inhibin A, estradiol, and Inhibin B.

	**Inhibin A**	**E2**	**Inhibin B**
Cycle day (*n*) *p*-value Pearson coefficient	*p* = 0.5933 0.04628 CI95% [−0.12; 0.21]	*p* = 0.0949 0.14381 CI95% [−0.03; 0.30]	*p* = 0.0137 −0.04921 CI95% [−0.21; 0.12]
Number of stimulation days *p*-value Pearson coefficient	*p* = 0.3056 0.08850 CI95% [−0.1; 0.25]	*p* = 0.0466 0.17095 CI95% [0.0; 0.32]	*p* = 0.8333 −0.01821 CI95% [−0.18; 0.15]
P4 level *p*-value Pearson coefficient	*p* < 0.0001 0.65165 CI95% [0.54; 0.73]	*p* < 0.0001 0.62761 CI95% [0.51; 0.71]	*p* < 0.0001 0.50450 CI95% [0.36; 0.62]
Total gonadotropin dosage *p*-value Pearson coefficient	*p* < 0.0001 −0.60 CI95% [−0.69; −0.47]	*p* < 0.0001 −0.47 CI95% [−0.60; −0.33]	*p* < 0.0001 −0.62 CI95% [−0.72; −0.51]
Number of retrieved oocytes *p*-value Pearson coefficient	*p* < 0.0001 0.82 CI95% [0.75; 0.86]	*p* < 0.0001 0.69 CI95% [0.60; 0.77]	*p* < 0.0001 0.71 CI95% [0.62; 0.78]
Number of mature oocytes *p*-value Pearson coefficient	*p* < 0.0001 0.77 CI95% [0.69; 0.83]	*p* < 0.0001 0.69 CI95% [0.59; 0.77]	*p* < 0.0001 0.65 CI95% [0.54; 0.74]

*E2: estradiol; P4, progesterone; CI95%, confidence interval*.

**Figure 2:**
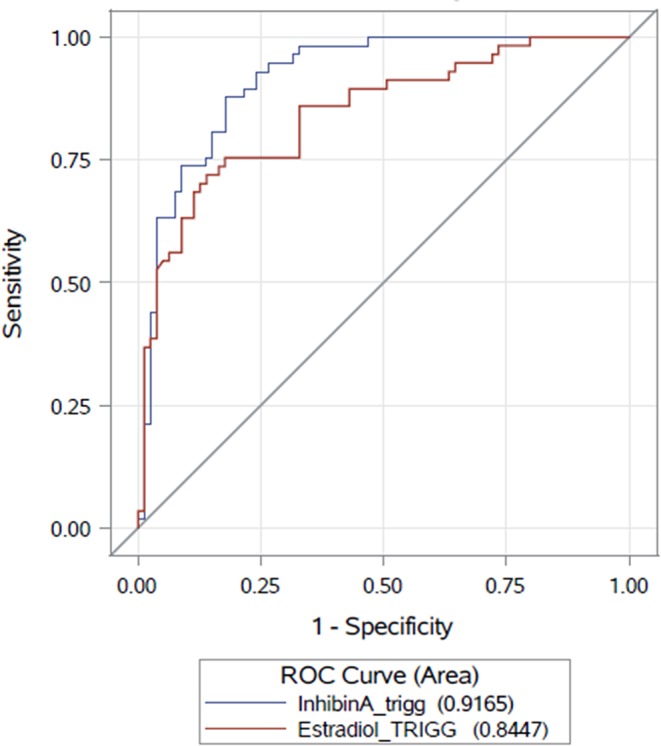
Correlations between Inhibin A/E2 and number of retrieved/mature oocytes as scatter plots.

A multivariate analysis was applied to evaluate the capacity of serum Inhibin A as a marker for retrieval of mature oocytes, controlling for age, and AFC on the day of final oocyte maturation. On the day of final oocyte maturation, serum Inhibin A level was found to be a predictive marker for the timing of final oocyte maturation (*p* < 0.0001) and was highly significantly correlated with the AFC (*p* < 0.0001), but not with age (*p* = 0.6473). The AUC for Inhibin A as a predictor for ≥10 mature oocytes was ρ 0.91 (CI95% [0.87; 0.96]) and ρ 0.84 (CI95% [0.7769; 0.9124]) for E2. The AUCs for both parameters are presented in Figure 2. The threshold level of serum Inhibin A level to predict the number of mature oocytes ≥10 (with an equivalent ratio of Sensitivity and Specificity) was 668.1 pg/mL (sensitivity = 88.0%, specificity = 82.0%).

In order to describe the mean, the SD (standard deviation), the 95% Confidence Interval (95%CI) as well as the ranges of the serum levels of Inhibin A and E2 depending on the number of ultrasonographic visible follicles on the day of final oocyte maturation, the follicle numbers were divided into groups and the results are presented in Supplementary Table 1. Supplementary Tables 2, 3 summarize the results of the descriptives of the serum levels of Inhibin A and E2, when analyzed according to the number of retrieved oocytes and mature oocytes.

## Discussion

To the best of our knowledge, this is the largest prospective observational, cross-sectional study, measuring the levels of Inhibin A in blood samples, obtained during ovarian stimulation for IVF/ICSI, in order to evaluate the efficacy of serum Inhibin A levels as a predictive parameter for the timing of final oocyte maturation in ovarian stimulation for IVF/ICSI, in comparison to serum E2 levels.

The findings of our study demonstrate, that on the day of final oocyte maturation Inhibin A is strongly correlated to the number of follicles ≥15 mm (ρ 0.72) and to the number of retrieved and mature oocytes (ρ 0.82/0.77, respectively), whereas E2 has only moderate correlations to the parameters mentioned (ρ 0.65/0.69/0.69, respectively). With an AUC of 0.91 for Inhibin A, compared to an AUC of 0.84 for E2, serum Inhibin A levels can be regarded as a better predictor of the optimal time for administration of trigger medication with a number of ≥10 mature oocytes. As a consequence of these data it can be concluded, that serum levels of Inhibin A may represent in combination with TVUS a more accurate hormonal indicator for retrieval of mature oocytes and may be a more powerful tool in the decision making process on trigger timing as compared to serum levels of E2. Hence, it has to be stated that the clinical significance of this difference will have to be demonstrated in future studies.

Inhibin B, which is also used as a quantitative ovarian reserve parameter (18), was confirmed to be highly statistically significantly (*p* < 0.0001) correlated with AFC on cycle day 2 or 3 of the cycle confirming its role in assessing the existing follicle pool. Inhibin B levels were seen to increase during hormonal stimulation as a result of multifollicular growth, which is in keeping with previous reports (19). A moderate correlation was seen between Inhibin B, as well as between E2, and the number of mature oocytes. On this basis it can be concluded that the introduction of Inhibin B monitoring into routine clinical practice on the day of final oocyte maturation will not be of added benefit.

These findings do not concur with the study of Eldar-Geva et al. (20), who described a better correlation of Inhibin B with oocyte number, compared to Inhibin A and E2. The differences in the study findings may be explained by a larger sample size in our study (136 vs. 38) and a different patient population as our patients were older and had a higher BMI. Stimulation durations were approximately the same in both studies. Eldar-Geva et al. (20) stimulated either with a daily dosage of 100 or 200 IU, whereas in our population the mean stimulation dosage was 333 IU/day. Analyzing the differences in stimulation parameters in both studies, it can be assumed, that the patient population in the study of Eldar-Geva et al. (20) was predominantly composed of “good-prognosis” patients, whereas our study included patients undergoing ovarian stimulation for IVF/ICSI regardless of their ovarian reserve, a study population more closely reflecting “daily life” in an IVF center.

When serum levels of Inhibin B, E2, and Inhibin A were measured on day 5 of ovarian stimulation in a GnRH-agonist protocol, results indicated that Inhibin B may be a useful marker of follicular activity from smaller follicles at the time of recruitment and selection. On stimulation day 5, Inhibin B, but not E2, was shown to directly correlate with ovarian response. In addition, day 5 Inhibin A serum level correlated with the number of mature follicles in the late follicular phase. These study findings may be explained by the increased sensitivity of Inhibin B to the FSH-stimulus in the early follicular phase of ovarian stimulation as compared to serum levels of Inhibin A and E2, leading to an earlier rise of Inhibin B in serum (21).

Cycle monitoring is, as previously mentioned, an essential part of ART treatment and serum E2 level monitoring has been the cornerstone of ovarian stimulation cycles with gonadotropins since IVF inception. In 1990, Hardiman et al. (22) critically analyzed the correlation between serum E2 levels and ultrasonographic follicular development and demonstrated that the concentration of E2 in serum as well as in saliva correlates better with the total follicle number rather than with the number of mature follicles. They concluded that E2 serum level is a poor indicator of follicular maturity compared to ultrasound findings. In the following years, more studies investigated the added benefit of E2 measurements to the outcome of IVF treatment: In an RCT (randomized controlled trial), Golan et al. (23) monitored patients, undergoing HMG stimulation in a GnRH-agonist protocol, either with ultrasonography and serum E2 measurements or with ultrasound in isolation. No significant differences were found between the two groups in terms of HMG stimulation duration, the total gonadotropin dosage, the number of oocytes retrieved and embryos transferred and the pregnancy rates. This study concluded that addition of E2 serum level measurements to ultrasound findings during cycle monitoring does not increase the number of mature oocytes retrieved. A further study by Vandekerckhove et al. (24) concurred with this study conclusion. Moreover, the theory that a certain E2-to-oocyte ratio at the time of hCG administration would result in improved implantation, pregnancy and live-birth (25) was refuted by Lass and UK Timing of hCG Group (26), who did not find any clinical benefit to including E2 measurement as a factor to consider when deciding when to administer hCG (human Chorionic Gonadotropin) for final oocyte maturation. The updated (6) Cochrane review (27), based on 6 instead of only 2 RCTs, did not find evidence for a better outcome by the addition of E2 in cycle monitoring. Despite the absence of conclusive evidence of a benefit to E2 monitoring, the Cochrane review recommended that a combined monitoring protocol including both, TVUS and serum E2, may need to be retained as precautionary good clinical practice.

Serum levels of E2 levels are often referred to as being an important marker for the prediction of ovarian hyperstimulation syndrome (OHSS) (28), which is a severe and possibly life-threatening complication of ovarian stimulation for IVF (29). However, OHSS can also occur in patients who conceived spontaneously or in patients with low serum E2 levels on the day of final oocyte maturation, thereby challenging the “E2 myth” in OHSS prediction (30). Further studies demonstrated that the number of developing follicles (31, 32) are superior to the E2 concentration on day of final oocyte maturation for identifying patients at risk for OHSS.

Crucial to the success of ART outcome is the number of mature oocytes, available for fertilization. Hence, cycle monitoring tools should consist of parameters with the capability of identifying the optimal time for final oocyte maturation.

Ultrasonographic measurement of the follicle number and size, is an essential part of cycle monitoring and several studies have been conducted to evaluate the “optimal” follicle size for triggering ovulation. Current literature suggests that follicles of sizes 16–22 mm on the day of oocyte retrieval contribute the most to the number of oocytes retrieved and that oocytes, derived from medium size follicles (13–23 mm), have equal maturity rates, fertilization rates, and blastocyst development compared to oocytes from large size follicles (≥23 mm). These rates were significantly lower for oocytes obtained from small follicles (8–12 mm) (33–35). Follicle growth is assumed to occur at a pace of ~1.7 mm/day, theoretically resulting in a follicle size at the time of trigger administration, 34–38 h prior oocyte retrieval procedure, of 12–18 mm. Abbara et al. (36) confirmed the follicle size of 12–19 mm on the day of trigger as the size which is most likely to yield mature oocytes.

Inhibins are produced by granulosa cells and exist as heterodimeric glycoproteins, composed of an α-subunit linked to either a ßA-subunit (Inhibin A) or a ßB-subunit (Inhibin B) (37). Together with E2, Inhibin A, and Inhibin B play a role in restraining FSH in order to ensure the development of a single dominant follicle (38). Inhibin B is mainly secreted by the smaller, non-selected cohort follicles, whereas Inhibin A is mainly produced by the dominant follicle and in a natural cycle, Inhibin A levels start to rise in the late follicular phase with the presence of larger size follicles of a size of ~15 mm (11, 39). Oocytes, derived from medium size follicles have equal maturity-, fertilization-, and blastocyst development rates as compared to oocytes from large size follicles.

An Inhibin A serum level of 668.1 pg/ml could be identified as a minimum threshold above which it is likely to retrieve ≥10 mature oocytes. In light of previous publications, evaluating the association between the number of retrieved oocytes and live birth (2)/cumulative live birth rates (40) and describing a number of retrieved oocytes to maximize live birth rate as ~15 (2)/and a relatively unchanged live birth probability between seven and 20 oocytes retrieved (40), respectively, the number of ≥10 mature oocytes presents a robust marker for a successful ART outcome. The only publication referring to the number of mature oocytes in the context of live birth rates derived their data from oocyte donation cycles (41). They described the threshold number as >10 oocytes retrieved, mature oocytes, zygotes, and cleaved embryos, as significant predictors of live birth as compared to <10 of each of these variables. Therefore, Inhibin A serum level represents an excellent parameter for the planning of trigger administration and oocyte retrieval in combination with TVUS.

The strength of our study lies in the sample size and the inclusion of patients independent of their quantitative ovarian reserve parameters, representing the “real life scenario” of an IVF center. Inhibin A has the potential to serve as a decision making tool when deciding the optimal time to trigger. Limitations of the study are that some of the included patients had previously performed stimulation cycles which may have influenced the decision when to administer medication for final oocyte maturation and that Inhibin A results were not taken into account when deciding on the trigger timing and therefore Inhibin A serum level was not used as a decision-making tool in this setting.

## Conclusions

The herein presented data revealed that serum levels of Inhibin A correlates better with the number of follicles ≥15 mm on the day of final oocyte maturation as well as with the number of retrieved and mature oocytes, compared to serum levels of E2. Therefore, serum Inhibin A levels may represent in combination with TVUS a promising tool for the planning of oocyte retrieval procedures. Currently there are no other studies demonstrating the predictive value of serum levels of Inhibin A on the number of mature oocytes. Large, prospective randomized studies are required to confirm our study findings and before the introduction of serum Inhibin A monitoring into routine clinical practice.

## Data Availability Statement

All datasets generated for this study are included in the article/Supplementary Material.

## Ethics Statement

The studies involving human participants were reviewed and approved by Ethics committee of IVIRMA Middle East Fertility Clinic Abu Dhabi, United Arab Emirates. The patients/participants provided their written informed consent to participate in this study.

## Author Contributions

BL: conceptualization of the study, data analysis, drafting, and review of paper. LD: statistical analysis and review of paper. CC: review of paper and linguistic revision of paper. CA and LM: review of paper. BK: immunoassay design and hormone measurements and review of paper. GS: immunoassay design and review of paper. HF and AK: conceptualization of the study and review of paper.

## Conflict of Interest

BK, GS, and AK are employees of Ansh Labs. They participated in the blinded analysis of the samples, however the final data collection and statistical analysis of the results, as well as the decision to publish was taken by BL, CA, and HF. The funder did not have any additional role in the study design, data collection and analysis, decision to publish, or preparation of the manuscript. The commercial affiliation of BK, GS, and AK did not play any role in the study. The remaining authors declare that the research was conducted in the absence of any commercial or financial relationships that could be construed as a potential conflict of interest.
