# Supplementary material for: Inhibin A—A Promising Predictive Parameter for Determination of Final Oocyte Maturation in Ovarian Stimulation for IVF/ICSI
Source: Front Endocrinol (Lausanne). 2020 May 15;11:307. doi: 10.3389/fendo.2020.00307 (PMC7243678; doi:10.3389/fendo.2020.00307)
Supplement: Supplementary file 1 [file Data_Sheet_1.docx]

**Supplementary table 1: Inhibin A- and estradiol-levels on the day of final oocyte maturation in groups with different follicle numbers**

| **Inhibin A (pg/ml) on the day of final oocyte maturation in groups with different follicle numbers** | | | | | | | |
| --- | --- | --- | --- | --- | --- | --- | --- |
| **groups** | **Number of samples** | **Mean** | **SD** | **95%CI** | | **Min** | **Max** |
|  |  |  |  | **Lower bound** | **Upper bound** |  |  |
| **< 5** | 14 | 132.7 | 88.7 | 81.5 | 184.0 | 10.6 | 339.6 |
| **5 - <10** | 28 | 347.3 | 158.8 | 285.7 | 408.9 | 115.9 | 636.1 |
| **10 - <15** | 32 | 646.1 | 272.0 | 548.0 | 744.2 | 285.0 | 1412.4 |
| **15 - <20** | 28 | 1000.2 | 373.7 | 855.3 | 1145.1 | 398.5 | 2225.8 |
| **≥ 20** | 34 | 1538.5 | 672.8 | 1303.7 | 1773.2 | 428.4 | 3859.5 |
| **E2 (pg/ml) on the day of final oocyte maturation in groups with different follicle numbers** | | | | | | | |
| **groups** | **Number of samples** | **Mean** | **SD** | **95%CI** | | **Min** | **Max** |
|  |  |  |  | **Lower bound** | **Upper bound** |  |  |
| **< 5** | 14 | 491.2 | 240.8 | 352.2 | 630.2 | 82.9 | 909.9 |
| **5 - <10** | 28 | 1020.7 | 496.3 | 828.3 | 1213.2 | 205.5 | 2102.3 |
| **10 - <15** | 32 | 1670.9 | 673.0 | 1428.3 | 1913.6 | 652.2 | 3656.0 |
| **15 - <20** | 28 | 2718.5 | 1227.9 | 2242.3 | 3194.6 | 1019.1 | 5546.7 |
| **≥ 20** | 34 | 3567.0 | 1907.1 | 2901.5 | 4232.4 | 723.2 | 9005.1 |

E2: estradiol; SD: Standard deviation; CI: Confidence intervall;

**Supplementary table 2: Inhibin A- and estradiol-levels on the day of final oocyte maturation in groups with different numbers of retrieved oocytes**

| **Inhibin A (pg/ml) on the day of final oocyte maturation in groups with different numbers of retrieved oocytes** | | | | | | | |
| --- | --- | --- | --- | --- | --- | --- | --- |
| **groups** | **Number of samples** | **Mean** | **SD** | **95%CI** | | **Min** | **Max** |
|  |  |  |  | **Lower bound** | **Upper bound** |  |  |
| **< 5** | 32 | 234.1 | 177.4 | 170.1 | 298.1 | 10.6 | 875.6 |
| **5 - <10** | 32 | 510.7 | 186.5 | 443.5 | 578.0 | 250.2 | 981.0 |
| **10 - <15** | 24 | 884.3 | 330.4 | 744.8 | 1023.9 | 428.4 | 1861.6 |
| **15 - <20** | 21 | 1020.3 | 311.7 | 878.4 | 1162.1 | 558.2 | 1476.4 |
| **≥ 20** | 27 | 1706.9 | 660.8 | 1445.5 | 1968.3 | 736.8 | 3859.5 |
| **E2 (pg/ml) on the day of final oocyte maturation in groups with different numbers of retrieved oocytes** | | | | | | | |
| **groups** | **Number of samples** | **Mean** | **SD** | **95%CI** | | **Min** | **Max** |
|  |  |  |  | **Lower bound** | **Upper bound** |  |  |
| **< 5** | 32 | 747.9 | 491.1 | 570.9 | 925.0 | 82.9 | 2468.5 |
| **5 - <10** | 32 | 1504.5 | 465.9 | 1336.5 | 1672.4 | 441.2 | 2102.3 |
| **10 - <15** | 24 | 2296.0 | 1675.7 | 1588.5 | 3003.6 | 652.2 | 9005.1 |
| **15 - <20** | 21 | 2606.9 | 1182.3 | 2068.8 | 3145.1 | 724.4 | 4492.3 |
| **≥ 20** | 27 | 3866.4 | 1682.8 | 3200.7 | 4532.1 | 723.2 | 7407.1 |

E2: estradiol; SD: Standard deviation; CI: Confidence interval

**Supplementary table 3: Inhibin A- and estradiol-levels on the day of final oocyte maturation in groups with different numbers of mature oocytes**

| **Inhibin A (pg/ml) on the day of final oocyte maturation in groups with different numbers of mature oocytes** | | | | | | | |
| --- | --- | --- | --- | --- | --- | --- | --- |
| **groups** | **Number of samples** | **Mean** | **SD** | **95%CI** | | **Min** | **Max** |
|  |  |  |  | **Lower bound** | **Upper bound** |  |  |
| **< 5** | 45 | 297.1 | 202.5 | 236.3 | 357.9 | 10.6 | 890.6 |
| **5 - <10** | 34 | 744.7 | 441.2 | 590.8 | 898.7 | 250.2 | 2614.3 |
| **10 - <15** | 31 | 1057.9 | 418.3 | 904.4 | 1211.3 | 428.4 | 2155.9 |
| **15 - <20** | 10 | 1208.7 | 411.9 | 914.1 | 1503.3 | 759.4 | 2145.5 |
| **≥ 20** | 16 | 18112.5 | 709.9 | 1434.2 | 2190.7 | 816.8 | 3859.5 |
| **E2 (pg/ml) on the day of final oocyte maturation in groups with different numbers of mature oocytes** | | | | | | | |
| **groups** | **Number of samples** | **Mean** | **SD** | **95%CI** | | **Min** | **Max** |
|  |  |  |  | **Lower bound** | **Upper bound** |  |  |
| **< 5** | 45 | 938.5 | 562.8 | 769.4 | 1107.5 | 82.9 | 2468.5 |
| **5 - <10** | 34 | 1939.0 | 1090.2 | 1558.6 | 2319.4 | 441.2 | 6857.5 |
| **10 - <15** | 31 | 2617.1 | 1576.4 | 2038.8 | 3195.3 | 652.2 | 9005.1 |
| **15 - <20** | 10 | 2893.5 | 1320.5 | 1948.9 | 3838.1 | 724.4 | 4509.1 |
| **≥ 20** | 16 | 4256.2 | 1766.8 | 3314.7 | 5197.6 | 723.2 | 7407.1 |

E2: estradiol; SD: Standard deviation; CI: Confidence interval
